# HIV self-testing adoption and post-test linkage to care among men who have sex with men in China: a nationwide cross-sectional survey

**DOI:** 10.1186/s12879-024-09419-5

**Published:** 2024-05-27

**Authors:** Fan Yang, Peizhen Zhao, Weiming Tang, Joseph D. Tucker, Wenqian Xu, Cheng Wang

**Affiliations:** 1 Institute of Population Research, Peking University, Beijing, China; 2 Dermatology Hospital of Southern Medical University, Guangzhou, China; 3 Southern Medical University Institute for Global Health, Guangzhou, China; 4 School of Medicine, University of North Carolina at Chapel Hill, Chapel Hill, USA; 5 London School of Hygiene and Tropical Medicine, London, UK; 6 School of Public Health, Southern Medical University, Guangzhou, China

**Keywords:** HIV self-testing, Implementation, Adoption, Men who have sex with men, China

## Abstract

**Background:**

HIV self-testing (HIVST) was recommended to improve HIV testing services. China initiated some of the first HIVST pilots in the world, providing a unique opportunity for implementation research. We aim to investigate HIVST adoption and its following linkage to care among Chinese men who have sex with men (MSM).

**Methods:**

Data were collected using an online questionnaire distributed on major social media platforms in 2018, one year after HIVST was officially endorsed and allowed for sale. MSM who were at least 16 years old, assigned as male at birth, and ever tested for HIV were eligible. Primary outcome, adoption was defined as ever use of HIVST. Bivariate and multivariable logistic regressions were performed to explore the association between HIVST adoption and sociodemographic and behavioral factors. Linkage to care was also described via the following sequential events as indicators: (1) receiving result after recent test (2), seeking care from healthcare facility if test result was positive or indeterminate, and (3) delayed time before seeking care.

**Results:**

A total of 540 participants were included with an average age of 27.4 ± 6.6. Most were never married (87.4%) and half completed college (52.2%). Overall, 75.2% had adopted HIVST. Self-test kits were commonly obtained from community-based organizations (54.4%) and from online (46.6%). HIVST adoption was positively associated with having college or higher education (OR = 1.66, 95%CI: 1.07–2.57), and negatively associated with age older than 30 (AOR = 0.52, 95%CI: 0.32–0.84). Adoption was not associated with other socio-demographic or behavioral factors. After receiving HIV-positive or indeterminate results, 25/25 of HIVST adopters sought care while 3 out of 7 (42.9%) non-adopters sought care (*p* < 0.001). Delays before seeking care were not significantly different between HIVST adopters compared to non-adopters (*P* = 0.366).

**Conclusion:**

Many MSM adopted HIVST shortly after its launch. Our findings suggested that HIVST linkage to care is promising among MSM in China. Integration of HIVST with other essential sexual health services is needed.

**Supplementary Information:**

The online version contains supplementary material available at 10.1186/s12879-024-09419-5.

## Introduction

Human immunodeficiency virus (HIV) testing presents a crucial entry point into HIV-related care such as pre-exposure prophylaxis (PrEP) initiation and HIV treatment. Globally, 21% of people living with HIV are still unaware of their serostatus [1]. Testing for HIV has improved in many settings with committed public health programs over years but can be further strengthened to reach the first 95% goal by 2025 [2, 3]. Self-testing of HIV (HIVST) was defined by the World Health Organization (WHO) as a process where a person collects their own specimen, “performs the test and interprets their results, when and where they want” [4, 5]. HIVST has proven largely acceptable among men who have sex with men (MSM), sex workers, and people who inject drugs in previous studies from both high- and low-and-middle-income countries [6, 7]. Advantages of HIVST, when compared to facility-based HIV testing, may include that: HIVST allows users to test alone and in privacy, which protects them from HIV-related stigma and unvoluntary disclosure of test results [6]; also, HIVST, when conducted at home, spares the time and resources spent on transportation to testing sites at heath facilities or community-based organizations (CBO) [8].

MSM in China bear disproportionate burden of HIV [9]. HIV incidence rate among MSM was estimated to be 3.4 per 100 person-years based on an open cohort recruited in 2017 and 2018 [10, 11]. In 2017, approximately 25.5% of new HIV infections were among MSM [10]. 86% of MSM were not aware of their HIV serostatus and 47% reported having not tested over the past 12 months [12]. One survey study conducted in Beijing and Nanning of China found that over 78% of MSM reported that hypothetically they would be interested in using HIVST [13]. This initial interest was augmented by increased access to HIVST through e-commerce platforms where rapid diagnostic testing kits are sold online at the cost of 16 to 80 Chinese Yuan (2.3 to 11.5 USD). Nevertheless, in contrast to the common interest and access is the relatively low uptake rate. For instance, one study reported that only 29% of MSM surveyed have ever used HIVST in 2015 [14]. That suboptimal adoption of HIVST embodied a “know-do gap” which warrants implementation research on HIVST adoption.

Implementation science focus on “research methods and strategies to improve the use of evidence-based interventions” in real-world settings [15, 16]. In implementation science, adoption can be understood as the process where a novel regimen or behavior becomes used by its target population [17, 18]. Research on adoption focuses on the associated factors related to adoption by comparing those who adopt and those who fail to adopt [17]. Similar focus, in community health theories, was placed on comparing “doers” and “non-doers” of certain health behavior, such as quitting smoking, or sleeping under an insecticide-treated bed net for cardiovascular disease and malaria prevention respectively [19, 20]. Such analytical design is also useful to shed light on human behavior about HIVST. HIVST adoption is hereafter defined operationally as lifetime uptake of HIVST. Identifying HIVST adopters and non-adopters among the target population, and understanding their characteristics can potentiate more equitable and comprehensive HIV services and outcomes.

Previous studies on HIVST largely concentrated on the extent to which users performed the test correctly on their own, and what common “user errors” were; several studies also explored behavioral interventions to promote HIVST in controlled settings [21–24]. To date, empirical studies on MSM’s adoption of HIVST remains scarce in low- and middle- income settings. Few study examined subsequent behaviors following the use of HIVST among Chinese MSM [25]. By guideline, HIVST serves as a test for triage and should be accompanied by provider-administered HIV testing when an initial positive or indeterminate result needs to be further confirmed [5]. Therefore, examining the linkage to subsequent care and services after HIVST is crucial to maximize the benefits brought by HIVST. Yet, no studies have reported, up to the present, on the linkage to care following a positive or indeterminate result among MSM in China.

In 2017, the State Council of China issued a policy memorandum “China AIDS Control, Prevention and Treatment Thirteen-Five Action Plan” which officially endorsed HIVST delivery through pharmacies and internet sales [26]. Ever since, various actors, such as MSM CBOs, research groups and online shops, have started advertising and providing HIVST kits in China. We conducted the present study one year after this major HIV policy milestone, aiming to fill the research gap on HIVST adoption in a natural setting, and investigate the subsequent linkage to care among Chinese MSM.

## Methods

### Study design

We designed and implemented a cross-sectional survey to collect information on HIV testing histories, results, and preferences among Chinese MSM across China [27]. Sociodemographic and behavioral data included age, marital status, education, income, and sexuality, sexual roles, alcohol and substance use, condom use, and disclosure of same-sex practices to family, friends, and healthcare providers. Questionnaire was pre-programmed on an online platform and then distributed via Chinese MSM online communities from July 14 to 28, 2018 [27]. Several CBOs helped advise on the sensitivity of questions and promote this survey in their social and professional network.

### Eligibility and recruitment

Eligibility includes: (1) age being 16 years or older; (2) assigned as male at birth; (3) ever had sex with another man; and (4) ever tested for HIV. For recruitment, we advertised the study on WeChat news feed, WeChat official account postings, and Weibo. Interested people could choose to click on a link to initiate an eligibility assessment. If eligible, they can then consent and proceed to complete the pre-programmed questionnaire online. For quality control purpose, each unique phone number could only be used once to participate in the survey, in order to prevent duplicated data submission from the same person. Each participant received 20 Chinese Yuan (about 3 in US Dollars) as compensation for their time after completing the survey.

### Measures

Primary outcome was HIVST adoption, a binary variable. Participants who reported having ever used HIVST by the time of the survey were categorized as HIVST adopters; if not, they were categorized as non-adopters. For adopters, the source of HIVST kits was also asked in a multiple-choice question (choices included online, hospital, pharmacy, CBO, friend, family, and others). Age was measured as a continuous variable and re-coded as binary in analysis (≤ 30, versus > 30); current marital status included 0 = never married, 1 = engaged or married, and 2 = separated, divorced or widowed; sexuality referred to self-identification as 0 = gay, 1 = bisexual, or 2 = none of above. Highest education attainment was categorical and recoded as binary (0 = less than college, 1 = college or above); individual monthly income measure was ordinal (six-tier). Participants who enrolled in full- or part-time education program at the time of survey were categorized as being current students (0 = no, 1 = yes). Also, participants were asked whether they disclosed sexuality to family or friends (0 = no, 1 = yes), and to healthcare providers (0 = no, 1 = yes).

Behavioral measures included during the past three months the sexual roles which participants primarily assumed (0 = insertive, 1 = receptive, 2 = versatile), condom use frequency (0 = never, 1 = sometimes used, 2 = mostly used, 3 = always used), condom use at the last sex with another man (0 = no, 1 = yes), and drunk sex (0 = no, 1 = yes). If participants were not sexually active during the past three months, they chose the response “not applicable” for those questions. For all participants, we also measured whether they had concurrent sexual partners, ever participated in group sex, had sex with female partners in the past six months, and ever used illicit drug during sex, all as dichotomous measures where 0 = no and 1 = yes. Two dichotomous variables measured bidirectional transactional sex: purchasing sex was defined as ever giving money or gifts to someone in order to have sex with them (0 = no, 1 = yes); selling sex was defined as ever receiving money or gifts to have sex with another person (0 = no, 1 = yes).

In terms of linkage to care following HIV testing, we compared HIVST adopters and non-adopters on the following indicators: (1) whether they received their most recent HIV test results, as an indication for awareness of HIV results through testing; (2) then, based on their recent HIV test results, to the sub-group who responded “positive/indeterminate” results for their last HIV test, we asked whether they had sought care at health facility (0 = no, 1 = yes); (3) last, another question asked about the time delay before seeking care once they knew their positive/indeterminate results, which was categorized into 0–2 weeks, 2–4 weeks, 1–3 months, longer than three months, and currently not in care).

### Statistical analysis

Bivariate and multivariable logistic regression analyses were conducted on Stata 17 to explore the associations between HIVST adoption and sociodemographic as well as sexual behavioral variables. In bivariate analysis, sociodemographic variables significantly associated with HIVST adoption were retained for multivariable regression analysis as covariates to be adjusted. In multivariable analysis, a total of ten models were built between each sexual or risk behavioral variable and HIVST adoption (as the outcome variable) respectively to assess their associations, controlling for sociodemographic covariates retained from previous bivariate analyses. Crude odds ratio (COR) and adjusted odds ratios (AOR) were calculated and reported with 95% confidence interval (CI). A significant level was defined at *P* < 0.10 for bivariate and *P* < 0.05 for multivariable analyses. For describing the linkage to care following indeterminate/positive results, univariate analyses were performed and yielded frequency and percentage for categorical variables, and mean, median, standard deviation (SD) and interquartile range (IQR) for continuous variables. Chi-square test was used to assess the difference in linkage to care comparing HIVST adopters to non-adopters. All analyses were pre-planned prior to accessing the dataset. We applied Two-sided Confidence Intervals (CI) for One Proportion method for sample size estimation, to have obtained a sample size of 305 for this study to produce a two-sided 95% CI and a width of 0.01. The current sample size of 540 has achieved this minimum requirement.

## Results

### Sociodemographic characteristics of study participants

A total of 540 participants were included in the analysis (see Table 1). Their mean age were 27.4 ± 6.6 years old. More than half of participants completed at least college or equivalent level of education (52.2%). Monthly income bracket mostly reported were between 3,100 and 5,200 Chinese Yuan (33.5%, 437 to 727 US Dollars), followed by 5,201-8,300 CNY (20.0%, 728–1164 USD). Participants were largely never married (87.4%), not currently students (74.6%), and identified as gay men (73.9%).

**Table 1:** Sociodemographic and behavioral characteristics of study participants (*N* = 540)

	Total *n* (%)	HIVST non-adopter *n* (%)	HIVST adopter *n* (%)	*p*-value
	*N* = 540	134, 24.8%	406, 75.2%	
***Sociodemographic variables***				
Age (years)				
Mean ± SD ≤30 >30	27.4 ± 6.6 408 (75.56) 132 (24.44)	28.4 ± 7.3 91 (67.91) 43 (32.09)	27.0 ± 6.3 317 (78.08) 89 (21.92)	**0.03** **0.02**
Education completed				
Less than college level College or above	258 (47.78) 282 (52.22)	73 (54.48) 61 (45.52)	185 (45.47) 221 (54.43)	**0.07**
Monthly income level				
<$218 $218–436 $437–727 $728–1164 $1165–1455 >$1455	80 (14.81) 88 (16.30) 181 (33.52) 108 (20.00) 43 (7.96) 40 (7.41)	24 (17.91) 17 (12.69) 52 (38.81) 23 (17.16) 7 (5.22) 11 (8.21)	56 (13.79) 71 (17.49) 129 (31.77) 85 (20.94) 36 (8.87) 29 (7.14)	0.251
Marital status				
Never married Engaged/married Other*	472 (87.41) 35 (6.48) 33 (6.11)	115 (85.82) 9 (6.72) 10 (7.46)	357 (87.93) 26 (6.40) 23 (5.67)	0.741
Being a student				
No Yes	403 (74.63) 137 (25.37)	105 (78.36) 29 (21.64)	298 (73.40) 108 (26.60)	0.253
Sexual orientation**				
Gay Bisexual None of above	399 (73.89) 115 (21.30) 26 (4.81)	97 (72.39) 30 (22.39) 7 (5.22)	302 (74.38) 85 (20.94) 19 (4.68)	0.898
Disclosing sexuality				
-To family/friends				
No Yes	292 (54.07) 248 (45.93)	68 (50.75) 66 (49.25)	224 (55.17) 182 (44.83)	0.373
-To healthcare workers				
No Yes	213 (39.44) 327 (60.56)	45 (33.58) 89 (66.42)	168 (41.38) 238 (58.62)	0.109
***Sexual behaviors***				
Sexual roles^†^				
Insertive Versatile Receptive Not applicable	183 (33.89) 87 (16.11) 183 (33.89) 87 (16.11)	39 (29.10) 24 (17.91) 47 (35.07) 24 (17.91)	144 (35.47) 63 (15.52) 136 (33.50) 63 (15.52)	0.574
Condom use at last sex^†^				
No Yes Not applicable	154 (28.52) 299 (55.37) 87 (16.11)	35 (26.12) 75 (55.97) 24 (17.91)	119 (29.31) 224 (55.17) 63 (15.52)	0.694
Condom use in general^†^				
Never used Sometimes used Mostly used Always used Not applicable	15 (2.78) 101 (18.70) 126 (23.33) 211 (39.07) 87 (16.11)	5 (3.73) 27 (20.15) 25 (18.66) 53 (39.55) 24 (17.91)	10 (2.46) 74 (18.23) 101 (24.88) 158 (38.92) 63 (15.52)	0.588
Concurrent sexual partners				
No Yes Missing	303 (56.11) 235 (43.52) 2 (0.37)	75 (55.97) 59 (44.03) 1 (0.75)	228 (56.16) 178 (43.84) 1 (0.25)	0.711
Ever participated in group sex				
No Yes	415 (76.85) 125 (23.15)	105 (78.36) 29 (21.64)	310 (76.35) 96 (23.65)	0.634
Had sex with female (last 6 months)				
No Yes Missing	485 (89.81) 49 (9.07) 6 (1.11)	121 (90.30) 12 (8.96) 1 (0.75)	364 (89.66) 37 (9.11) 5 (1.23)	0.895
Had drunk sex^†^				
No Yes Not applicable	303 (56.11) 150 (27.78) 87 (16.11)	76 (56.72) 34 (25.37) 24 (17.91)	227 (55.91) 116 (28.57) 63 (15.52)	0.690
Ever used illicit drug in sex				
No Yes	301 (55.74) 239 (44.26)	82 (61.19) 52 (38.81)	219 (53.94) 187 (46.06)	0.143
Ever bought sex				
No Yes	458 (84.81) 82 (15.19)	116 (86.57) 18 (13.43)	342 (84.24) 64 (15.76)	0.514
Ever sold sex				
No Yes	475 (87.96) 65 (12.04)	122 (91.04) 12 (8.96)	353 (86.95) 53 (13.05)	0.206

*Other: separated, divorced, or widowed

** None of above: including self-identifying as straight, unsure and other

† Time frame: past three months; 87 participants reported no sexual activities and therefore skipped these questions

### Sexual and behavioral risk factors

More than half (60.6%) had disclosed their sexuality to clinicians, but less (45.9%) did so to family or friends. Among all, 87/540 (16.1%) participants reported not being sexually active in the past three months. 299/540 (55.4%) reported being sexually active and used a condom during the last sexual intercourse; however, when it comes to consistent condom use over the past three months, this proportion declined to 39.1% (211/540). Among the 453 sexually active participants, 183/453 (40.4%) of them assumed receptive or mostly receptive role during intercourse, 87/453 (19.2%) versatile, and 183/453 (40.4%) insertive or mostly insertive.

Regarding other distal risk behaviors related to HIV, 43.5% (235/540) reported having had concurrent sexual partners; 23.2% (125/540) reported having ever participated in group sex; 27.8% (150/540) reported having ever had drunk sex; 44.3% (239/540) had used substances during sex before. Substances commonly mentioned by those participants included opioid-related drugs, marijuana, methamphetamine, and several other psychedelic chemicals. About 9.1% (49/540) of the total sample reported also having had sex with female sexual partners in the past six months. On transactional sex, responses showed that 15.2% (82/540) of our study sample ever bought sex while 12% (65/540) ever sold sex.

### HIVST adoption and associated factors

Three quarters of participants (75.2%, 406/540) reported that they have already used HIVST at the survey time one-year following the HIVST launch in China. In comparing HIVST adopters and non-adopters using chi-square test, adopters (mean age: 27.0) overall tend to be significantly younger than non-adopters (mean age: 28.4, *p* = 0.03). Besides, adopters (54.4%) tend to have a greater proportion that completed at least college or equivalent education, compared to non-adopters (45.5%, *p* = 0.07). Marital status, current student status, and other sociodemographic variables did not differ significantly between HIVST adopters and non-adopters. In bivariate analyses, the associations between HIVST adoption and age unfolded as followed: each year of increase in age was associated with 3% less likelihood of adopting HIVST. When using 30 years old as an age threshold, men who were older than 30 were 41% less likely to have adopted HIVST as a modality of testing (COR: 0.59, 95%CI: 0.39–0.92), compared to men who were 30 or younger. When it came to HIVST adoption and education attainment, one unit increase in education level was found to be positively associated with increased likelihood of HIVST adoption (COR: 1.41, 95%CI: 1.02–1.96).

Monthly income level, marital status, current student status, self-identified sexuality, and disclosure of sexuality did not appear to be significantly associated with HIVST uptake (See Table 2). Sexual behavioral and risk factor variables, including sexual roles, condom use behaviors, concurrent sexual partnership, group sex, drunk sex, illicit drug use during sex, transactional sex, and having female sexual partners, were not significantly associated with MSM’s HIVST uptake with 95%CIs spanning across 1.0 and *p*-value greater than 0.10.

**Table 2:** Bivariate analyses of self-testing adoption in relation to participants’ sociodemographic and behavioral characteristics (*N* = 540)

	Crude Odds Ratio	95% Confidence Interval	*p*-value
*Sociodemographic*			
Age (one unit increase in years)	**0.97**	**0.94- 1.00**	**0.029**
Age category			
≤30	1		
>30	**0.59**	**0.39–0.92**	**0.018**
One unit increase in education level	**1.41**	**1.02–1.96**	**0.037**
Monthly income level			
<$218	1		
$218–436	1.79	0.88–3.65	0.11
$437–727	1.06	0.60–1.89	0.835
$728–1164	1.58	0.82–3.08	0.175
$1165–1455	2.2	0.86–5.64	**0.099**
>$1455	1.13	0.49–2.62	0.776
Marital status			
Never married	1		
Engaged/married	0.93	0.42–2.04	0.858
Other*	0.74	0.34–1.60	0.446
Being a student			
No	1		
Yes	1.31	0.82–2.09	0.254
Sexual orientation**			
Gay	1		
Bisexual	0.91	0.57–1.46	0.697
None of above	0.87	0.36–2.14	0.764
Disclosing sexuality			
- To family/friends			
No	1		
Yes	0.72	0.48–1.08	0.11
-To clinicians			
No	1		
Yes	0.84	0.57–1.24	0.373
*Sexual behaviors*			
Sexual roles^†^			
Insertive	1		
Versatile	0.71	0.39–1.28	0.256
Receptive	0.78	0.48–1.27	0.325
Condom use at last sex^†^			
No	1		
Yes	0.88	0.56–1.39	0.58
Condom use in general^†^			
Never used	1		
Sometimes used	1.37	0.43–4.37	0.595
Mostly used	2.02	0.63–6.44	0.235
Always used	1.49	0.49–4.56	0.484
Concurrent sexual partners			
No	1		
Yes	1	0.68–1.49	0.985
Ever participated in group sex			
No	1		
Yes	1.12	0.70–1.80	0.634
Had sex with female (last 6 months)			
No	1		
Yes	1.02	0.52–2.03	0.944
Had drunk sex†			
No	1		
Yes	1.14	0.72–1.81	0.573
Ever used illicit drug in sex			
No	1		
Yes	1.35	0.90-2.00	0.143
Ever bought sex			
No	1		
Yes	1.05	0.92–1.19	0.493
Ever sold sex			
No	1		
Yes	1.1	0.97–1.25	0.153

*Other: separated, divorced, or widowed

** None of above: including self-identifying as straight, unsure and other

† Time frame: past three months; 87 participants reported no sexual activities and therefore skipped these questions

In multivariable regression analyses of HIVST adoption on pre-selected ten behavioral variables of interest (See Table 3), we found that none of them was significantly associated with HIVST adoption, after adjusting for age and education which were retained in multivariable models as covariates based on previous bivariate analysis findings. In all ten models built, age older than 30 years old was consistently associated with less likelihood of HIVST adoption with an AOR ranging from 0.52 to 0.62 and the upper bound of 95%CI below 1.0. Additionally, education level remained a statistically significant predictor for HIVST uptake, regardless of sexual roles, condom use at last sex, condom use frequency in the past three months, and drunk sex. Once adjusting for behavioral covariates respectively such as concurrent sexual partnership, group sex, sex with female partner, illicit drug use, or transactional sex, the education attainment was only marginally associated with HIVST adoption in multivariable regression models.

**Table 3:** Multivariable analyses of HIV self-testing adoption in relation to participants’ behavioral characteristics, age (> 30 years old versus 30 years old or younger) and education attainment (*N* = 540)

	Behavioral variables			Age > 30 years old v. ≤ 30 years old		Completed college v. other	
	(in column on the left)						
**Model 1 to 10**	AOR	95% CI	*p*-value	AOR	95%CI	AOR	95%CI
1. Sexual roles^†^							
Insertive	1			0.52**	0.32–0.84	1.66*	1.07–2.57
Versatile	0.73	0.40–1.33	0.306				
Receptive	0.74	0.45–1.22	0.239				
2. Condom use at last sex							
No	1			0.54**	0.34–0.86	1.68*	1.09–2.61
Yes	0.9	0.56–1.43	0.644				
3. Condom use frequency^†^				0.55*	0.34–0.88	1.67*	1.08–2.59
Never used	1						
Sometimes used	1.47	0.45–4.78	0.525				
Mostly used	1.94	0.60–6.31	0.269				
Always used	1.51	0.48–4.70	0.479				
4. Concurrent sexual partners				0.59*	0.38–0.91	1.42	0.96–2.11
No	1						
Yes	1.03	0.69–1.54	0.884				
5. Ever participated in group sex				0.59*	0.38–0.91	1.4	0.94–2.07
No	1						
Yes	1.18	0.74–1.92	0.481				
6. Had sex with female in past 6 months				0.60*	0.39–0.92	1.42	0.96–2.12
No	1						
Yes	1.03	0.52–2.06	0.927				
7. Ever had drunk sex†				0.54**	0.34–0.86	1.69*	1.09–2.61
No	1						
Yes	1.14	0.72–1.83	0.575				
8. Ever used illicit drug in sex				0.62*	0.40–0.96	1.41	0.95–2.09
No	1						
Yes	1.28	0.85–1.91	0.236				
9. Ever bought sex				0.60*	0.39–0.93	1.39	0.94–2.07
No	1						
Yes	1.18	0.67–2.09	0.57				
10. Ever sold sex				0.61*	0.40–0.95	1.4	0.94–2.07
No	1						
Yes	1.44	0.74–2.79	0.284				

† Time frame: past three months; 87 participants reported no sexual activities and therefore skipped these questions

*p < 0.05, ** *p* < 0.01

Note: In the rows, each behavioral variable of the ten behavioral variables (listed on the left) was assessed in a logistic model as a potential predictor for HIV self-test adoption, adjusting for age and education (of which the AOR are listed in the two columns on the right)

### Sources of HIVST kits among HIVST adopters

**Fig. 1:**
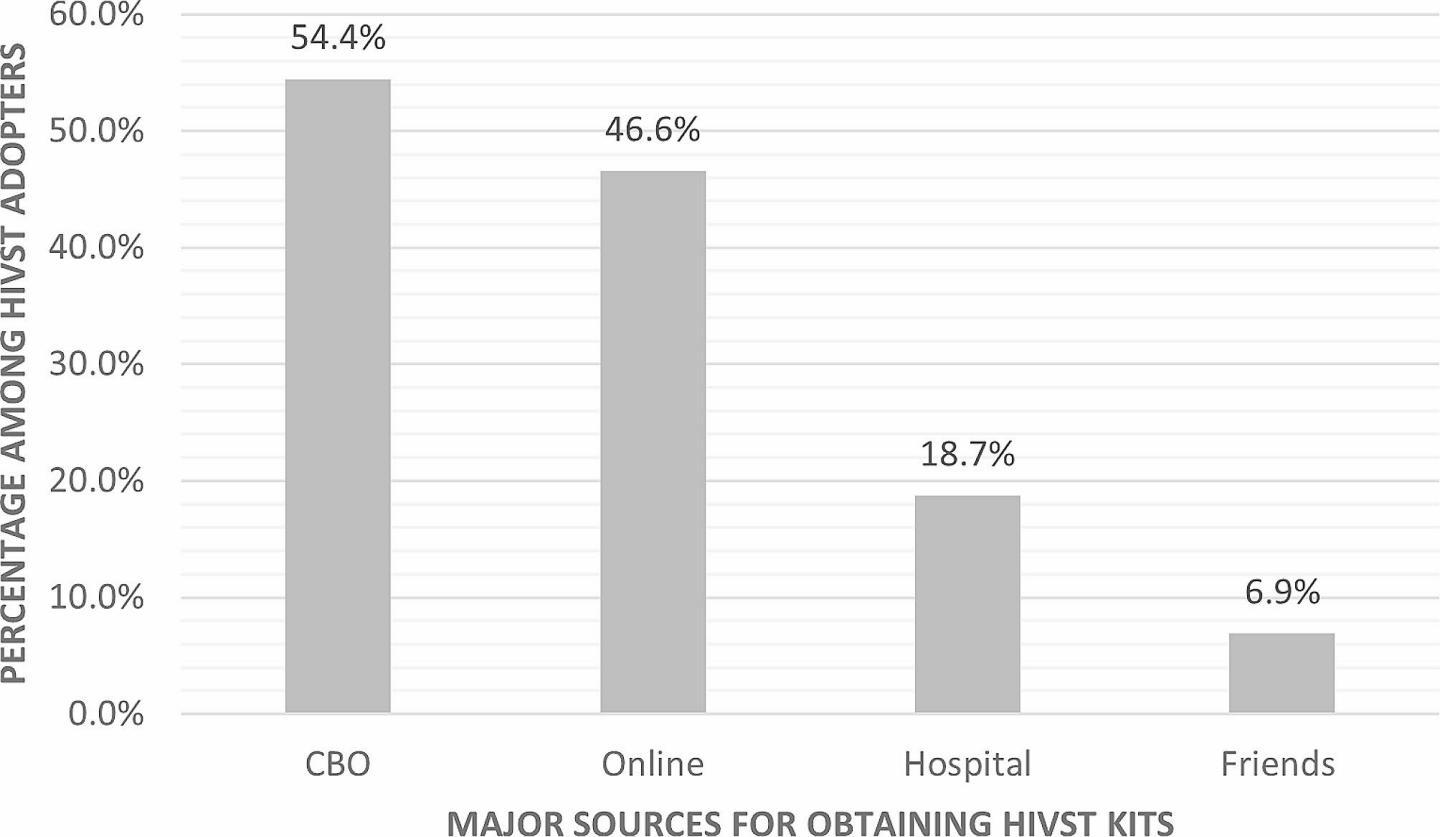
Source of HIVST kits reported by HIVST adopters (*N* = 406). Notes: categories are not mutually exclusive, HIVST adopters can select multiple sources where they obtained HIVST kits if applicable

Among HIVST adopters (*n* = 406), the two most common sources whereby participants obtained their HIVST kits were local CBOs (221/406, 54.4%) and online shops (189/406, 46.6%). Some also reported having obtained HIVST kits from hospitals (76/406, 18.7%) or from friends (28/406, 6.9%). None of the study participants ever obtained their HIVST from pharmacies or family members (see Fig. 1).

### Linkage to care comparing HIVST adopters versus non-adopters

Most participants reported having received their test results from the most recent testing, which did not significantly differ between HIVST adopters (96.3%) and non-adopters (92.5%, *p* = 0.07) from chi-square results (See Table 4). Among those participants who received positive or indeterminate HIV results, 25/25 adopters reported having sought care from health professionals, while only 42.9% of those HIVST non-adopters did (*p* < 0.001). Both HIVST adopters and non-adopters experienced time delay before seeking healthcare. That is, only 14.29% of adopters and 28.00% adopters sought care within 2 weeks after knowing their indeterminate or positive test results. The difference among adopters and non-adopters in term of time delays was not statistically significant different although the sample sizes for these measures were rather small (*P* = 0.366).

**Table 4:** Linkage to care after receiving a reactive result among HIVST adopters and HIVST non-adopters

	HIVST non-adopters	HIVST adopters	*p*-value
	n (%)	n (%)	
Received HIV results after most recent test	(N = 134)	(N = 406)	
No	10 (7.46)	15 (3.69)	0.072
Yes	124 (92.54)	391 (96.31)	
Among those receiving reactive/indeterminate results	(N = 7)	(N = 25)	
Sought care			
No	4 (57.14)	0 (0.00)	**0**
Yes	3 (42.86)	25 (100.00)	
Time before seeking care			
0–2 weeks	1 (14.29)	7 (28.00)	
2–4 weeks	2 (28.57)	6 (24.00)	0.366
1–3 months	1 (14.29)	5 (20.00)	
>3 months	1 (14.29)	6 (24.00)	
Currently not in care	2 (28.57)	1 (4.00)	

## Discussion

### Rapid adoption of HIVST and linkage to subsequent services

Overall, our study revealed that HIVST had gained substantial utilization among Chinese MSM shortly after its widespread introduction. Many MSM had already embraced HIVST as a testing method, with CBOs and online shopping platforms as primary sources for early adopters to access their HIVST kits. Our study confirmed that CBOs and online communities played an irreplaceable role in HIV service delivery not only in Jiangsu province, but also nationwide [28]. The adoption rate at three quarters from our study is much greater than previously reported adoption rate at 37.5% among Chinese MSM who tested negative for HIV [29]. Nevertheless, our findings added to existing empirical evidence suggesting that natural diffusion of HIVST in real world setting is unlikely to be as comprehensive as those reported in interventional studies. To in order to reach a wider coverage and provide full access to HIVST, intensified promotion of HIVST still needs to be in place.

Our findings also suggest that the adoption of HIVST does not pose additional challenges for linkage to care, thereby providing evidence to enhance the understanding of linkage to care following HIVST. Linkage to care is crucial in the context of self-testing, and scholars have proposed using linkage to care as an endpoint to measure the successful implementation of HIVST when self-testing modality progressed from clinical trial phases to real-world implementation [30]. Our study discovered that all HIVST adopters with reactive or indeterminate test results were connected to healthcare services after receiving their test results, and in fact, they demonstrated even higher rates of linkage compared to their non-adopter counterparts. Previously, most studies on linkage to care following HIVST have been concentrated in Sub-Saharan Africa and have yielded inconsistent results [31]. A prior study conducted among Chinese MSM found that only five sought care among the total 30 MSM who received reactive or indeterminate self-test results [32]. However, our findings, although derived from a relatively small sample, may suggest the opposite and add to the ongoing debate regarding potentially missed opportunities for linkage to care caused by the self-testing modality [30, 33]. It is important to note that further research is warranted to validate these observations in specific context.

### Demographic and economic factors in relation to HIVST adoption

Distinct demographic characteristics were observed among HIVST adopters. Notably, those who had adopted HIVST tended to be younger in age and possessed higher levels of education compared to those who had not utilized it at the time of the survey. These findings pointed to the demographic disparity that was often overlooked in epidemiological studies. In that sense, our findings contributed to a neglected area where age and education compounded the implementation of HIV preventive service as innovative modality of testing diffused into the market. One potential reason is that most HIV interventions in China are tailored to young MSM. Those programs, although reflect young people’s service needs, may neglect older and less educated subgroups. Future programs should purposefully reach and engage hidden subgroups within the MSM communities in co-creating HIV preventive messages and services. To increase HIVST uptake in specific older age group and low-education group, evidence-based behavioral interventions may be considered, such as secondary distribution of HIVST through MSM social network [34].

Our study also revealed that individual income brackets did not significantly influence the adoption of HIVST, which may seem surprising but is justifiable within China’s local HIV service delivery system. Financial barriers usually limited the access to self-testing despite the widespread interest in the novel testing modality [35]. For instance, mainstream brand kits for HIVST in the US were priced at approximately 40 USD at pharmacies and the high price excluded MSM with economic disadvantages [36]. In China, HIV services involved three levels of providers: key population-led CBOs, local centers for disease control (CDC), and designated hospitals specializing in infectious diseases in each city [37]. CBOs play a pivotal role, offering free initial HIV screening test, and referring those with reactive results to local CDCs for confirmatory testing [37]. From local CDCs, patients diagnosed with HIV infection are referred to treatment free of charge at designated hospitals. CBOs are the primary source of HIV testing for MSM, providing free testing services [37, 38]. When CBOs started offering free HIVST kits, economic barriers to HIVST adoption may become insignificant. This support from CBOs may have led to the seemingly equitable adoption of HIVST among MSM of different income levels during the study period. This finding highlights the effective synergy between different entities in China’s HIV service delivery system and its positive impact on HIVST diffusion among MSM, regardless of income levels. However, this HIVST delivery approach has also shifted the financial burden from individuals to the CBOs themselves [37, 38]. Unfortunately, many CBOs are increasingly facing budget cuts and reduced funding from the local CDCs [37, 38]. Considering this context, future research should closely monitor service delivery and reassess the financial sustainability of the current HIVST implementation [39].

### Sexual behavioral variables and their lack of association to self-testing modality

Our study revealed that high-risk sexual behaviors, such as concurrent sexual partners, no longer play a significant role in the adoption of HIVST. This finding contrasts with previous literature, suggesting that testing for HIV and the adoption of a novel testing modality are driven by different factors. In the context of innovation diffusion, the adoption of HIVST depends more on individual characteristics like age and education rather than perceived risks based on recent high-risk exposures [40].

Before our study, previous research on HIVST mainly focused on sexually active MSM, regardless of their HIV testing history [28, 41]. These studies compared HIVST users (cases) with non-users (controls), while controls included a mixture of individuals who had never tested for HIV, and those who had tested but not through self-testing [28, 41]. Consequently, such comparisons mixed the predictor variables for HIV testing in general with those for self-testing, leading to associations of HIVST ever-use with factors like marital status and having six or more concurrent sexual partners in the past six months [28, 41]. These behavioral factors may have encouraged HIV testing through perceived HIV risk enhancement but provided little information on the motivational factors specific to using self-testing. In our study, both HIVST adopters and non-adopters were MSM who had already tested for HIV, allowing us to isolate the adoption of the self-testing modality itself from general HIV testing behavior. This disentanglement provided insights into the distinct drivers of HIVST adoption, emphasizing individual characteristics rather than recent high-risk exposures.

### Limitations and strengths

Our study has several limitations. Firstly, its cross-sectional design restricted our ability to capture the full diffusion process of HIVST across various stages. Instead, we focused on a specific time point, one year after a major policy shift, to study HIVST adoption. While investigating natural diffusion within a cohort over time would be ideal, conducting long-term follow-ups with repeated surveys on HIVST could inadvertently promote HIVST through increased awareness, making the observational study less natural. To assess the natural diffusion of HIVST and similar HIV prevention methods, future studies could consider using a repeated cross-sectional design with non-overlapping samples. Secondly, our study has a limited scope, as it only characterizes HIVST adopters and non-adopters without offering specific strategies to engage non-adopters in HIVST implementation. To address this gap, further research is necessary, utilizing formative research to pilot strategies that promote inclusivity in HIVST adoption. Thirdly, our study sample was obtained through internet-based convenience sampling, which may limit the generalizability of our findings. For example, it is highly plausible that the influence of using the internet as a platform for recruitment is a significant factor contributing to the high HIVST adoption rate. While this sampling approach provided us access to a diverse range of participants, caution must be taken when extrapolating the results to broader populations. Additionally, because study required participants to register a phone number, individuals who were concerned about potential risks related to disclosing phone number, might opt out of participation, leading diminished representativeness of study sample.

This study exhibits several notable strengths. Firstly, the analysis was guided by implementation sciences theories and focused on adoption, a critical aspect of implementation which has been frequently overlooked [42]. Secondly, the sample used in this study covered three major geographic regions in China, offering a diverse representation of the population. Furthermore, data collection occurred at a crucial time window after a significant policy change, providing valuable insights into the effects of this change on HIVST adoption rates. To maintain data integrity, strict quality control measures were implemented during data collection. These measures effectively prevented duplicated participation and augmented the reliability and accuracy of the data collected.

Stigma surrounding HIVST can arise from misconceptions that HIVST users are more likely to be engaging in high-risk behavior. Contrary to these beliefs, our findings revealed that both HIV self-testing adopters and non-adopters exhibit similar levels of risk-taking behaviors. This contributes evidence to the field of HIV prevention by challenging existing assumptions and stereotypes about preventive measures and risk behaviors.

## Conclusion

Taken together, HIVST adoption was common among Chinese MSM and significantly associated with young age and high education, but not with any risky sexual behaviors. As HIVST continues to play a vital role in the fight against HIV/AIDS, understanding the factors influencing its adoption is essential in formulating effective public health strategies and interventions to maximize its impact in curbing the spread of HIV in China.

### Electronic supplementary material

Below is the link to the electronic supplementary material.

Supplementary Material 1

## Data Availability

De-identified data are available upon request to the corresponding author Cheng Wang.

## Abbreviations

AOR: Adjusted Odds Ratios
CBO: Community-Based Organizations
CDC: Centers for Disease Control
COR: Crude Odds Ratio
HIV: Human Immunodeficiency Virus
HIVST: HIV Self-Testing
IQR: Interquartile Range
MSM: Men who have Sex with Men
SD: Standard Deviation
